# Revision Surgery After Failed Fixation of Periprosthetic Distal Femur Fractures: Nail–Plate Combination Versus Double Plating

**DOI:** 10.3390/medicina62020275

**Published:** 2026-01-28

**Authors:** Bekir Karagoz, Hunkar Cagdas Bayrak, Tolga Kececi, Ali Okan Tarlacik

**Affiliations:** 1 Department of Orthopedics and Traumatology, Eskisehir City Hospital, 26080 Eskisehir, Turkey; 2 Department of Orthopedics and Traumatology, Cekirge State Hospital, 16090 Bursa, Turkey; 3 Department of Orthopaedics and Traumatology, Ordu University Training and Research Hospital, 52200 Ordu, Turkey

**Keywords:** periprosthetic fracture, distal femur fracture, revision surgery, nail-plate combination, double-plate fixation, technical trick

## Abstract

*Background and Objectives*: The aim of this study was to compare the clinical and radiological outcomes of the nail-plate combination (NPC) and double-plate (DP) fixation techniques in revision surgery performed after fixation failure of periprosthetic distal femur fractures. *Materials and Methods*: Patients who underwent revision surgery for periprosthetic distal femur fractures following fixation failure between 2018 and 2023 at a tertiary referral center were retrospectively reviewed. Based on the surgical technique, patients were divided into two groups: NPC group (*n* = 27) and DP group (*n* = 45). Demographic characteristics, operative time, intraoperative blood loss, and fluoroscopy time were recorded. Radiological evaluation included union time, while clinical outcomes were assessed with the Knee Society Score (KSS), Western Ontario and McMaster Universities Osteoarthritis Index (WOMAC), and the Short Form-36 (SF-36) health survey. Complications (infection, thromboembolism, implant failure, nonunion, malalignment), reoperation, and 1-year mortality rates were also analyzed. *Results*: The NPC group had significantly shorter operative time (107 vs. 134 min, *p* < 0.001) and lower intraoperative blood loss (412 vs. 634 mL, *p* < 0.001). Hospital stay was shorter in the NPC group (6.9 ± 1.5 vs. 10.2 ± 3.3 days, *p* < 0.001). Mean union time was approximately three weeks shorter in the NPC group (15.4 vs. 18.8 weeks, *p* < 0.001). Functional outcomes (KSS, WOMAC, SF-36) did not differ significantly between groups. Complication rates were comparable; implant failure was the most frequent complication (NPC: 3.7% vs. DP: 13.3%). One-year mortality did not differ significantly (NPC: 7.4% vs. DP: 11.1%). *Conclusions*: Compared with DP fixation, the NPC technique offers clear perioperative advantages in revision surgery performed after fixation failure of periprosthetic distal femur fractures, including shorter operative time, reduced blood loss, and faster union. Functional outcomes and complication rates were similar between techniques. These findings suggest that the NPC may represent a safer and more feasible alternative.

## 1. Introduction

Periprosthetic distal femur fractures are among the most common complications following total knee arthroplasty and are strongly associated with substantial morbidity and mortality, particularly in elderly patients with poor bone quality and multiple comorbidities [1,2]. With demographic shifts and the increasing number of knee arthroplasties being performed, the incidence of these fractures continues to rise [3]. This clinical scenario remains a challenging problem for both surgeons and patients, with demanding treatment strategies and high complication rates.

The primary goal of surgical management is to achieve stable fixation that permits early mobilization and reduces the risk of complications. However, consensus on the optimal fixation method has not yet been established. Double-plate (DP) fixation is frequently favored in unstable fractures or those with metaphyseal–diaphyseal extension due to the high rigidity it provides. Nevertheless, this technique is associated with certain drawbacks, including longer operative time, greater intraoperative blood loss, extensive soft tissue dissection, and, consequently, increased complication rates [1,4,5]. In contrast, combining an intramedullary nail with a lateral plate merges the load-sharing advantage of nailing with the supplemental support of plating, offering a biomechanically favorable construct with the potential for a less invasive surgical approach [6,7]. As such, this method has gained increasing attention in recent years.

Despite the growing body of literature on primary periprosthetic distal femur fractures, the literature addressing revision surgery after fixation failure in periprosthetic distal femur fractures remains limited. In particular, studies that directly compare the nail-plate combination (NPC) with DP fixation and evaluate perioperative, radiological, and functional outcomes are exceedingly scarce [8,9]. This knowledge gap continues to create uncertainty in surgical decision-making.

The aim of the present study was to compare the clinical, radiological, functional, and complication outcomes of NPC versus DP fixation in patients undergoing revision surgery for periprosthetic distal femur fractures following fixation failure. By focusing specifically on revision surgery, this study addresses a notable gap in the current literature and provides an innovative contribution that may guide surgical strategy.

## 2. Materials and Methods

### 2.1. Study Design and Patient Selection

**Figure 1:**
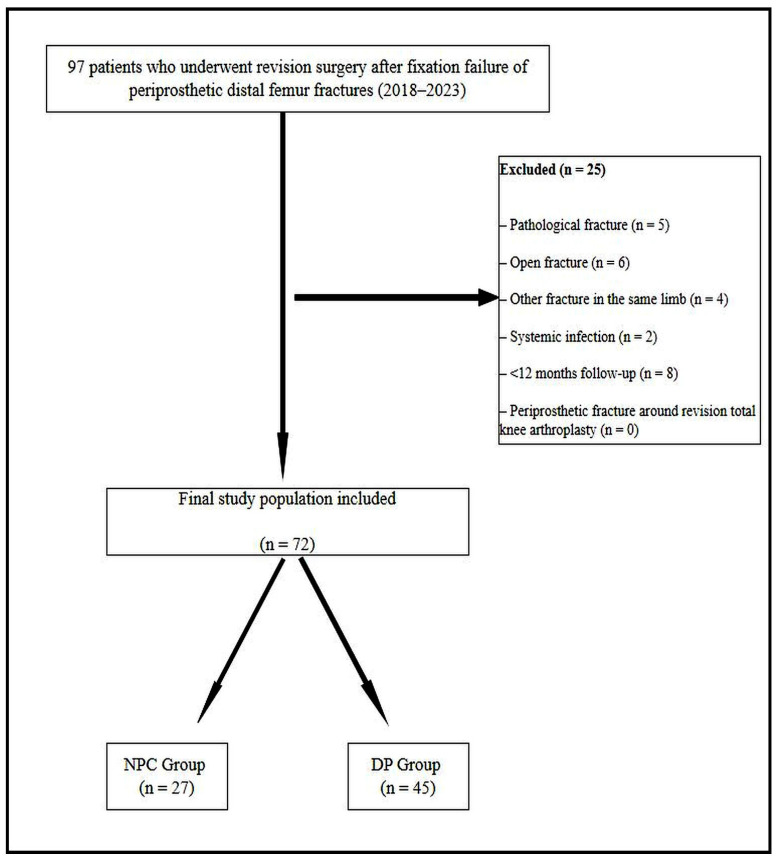
Flow diagram of patient inclusion and exclusion.

This study was designed as a retrospective cohort analysis. Institutional review board approval was obtained (Approval No: ESH/BAEK 2025/220, Date: 11 September 2025), and all procedures were conducted in accordance with the Declaration of Helsinki. Due to the retrospective nature of the study, the requirement for informed consent was waived by the institutional review board. Consecutive patients who underwent revision surgery following fixation failure of a periprosthetic distal femur fracture treated with osteosynthesis between 2018 and 2023 were evaluated. Ninety-seven patients were initially identified. Inclusion criteria were: (i) distal femur fracture after total knee arthroplasty treated surgically with a stable prosthetic implant, (ii) subsequent fixation failure requiring revision surgery, (iii) revision performed using either NPC or DP fixation, and (iv) availability of at least 12 months of clinical and radiological follow-up. Exclusion criteria included pathologic fractures, open fractures, concomitant ipsilateral fractures, systemic infection, periprosthetic fractures around revision total knee arthroplasties, and follow-up shorter than 12 months. After applying these criteria, 25 patients were excluded, and 72 consecutive patients remained eligible for analysis (Figure 1). Patients were classified into two groups based on the revision technique used: the NPC group (*n* = 27), treated with retrograde intramedullary nail combined with a lateral locking plate, and the DP group (*n* = 45), treated with medial and lateral locking plates.

### 2.2. Surgical Technique and Postoperative Care

**Figure 2:**
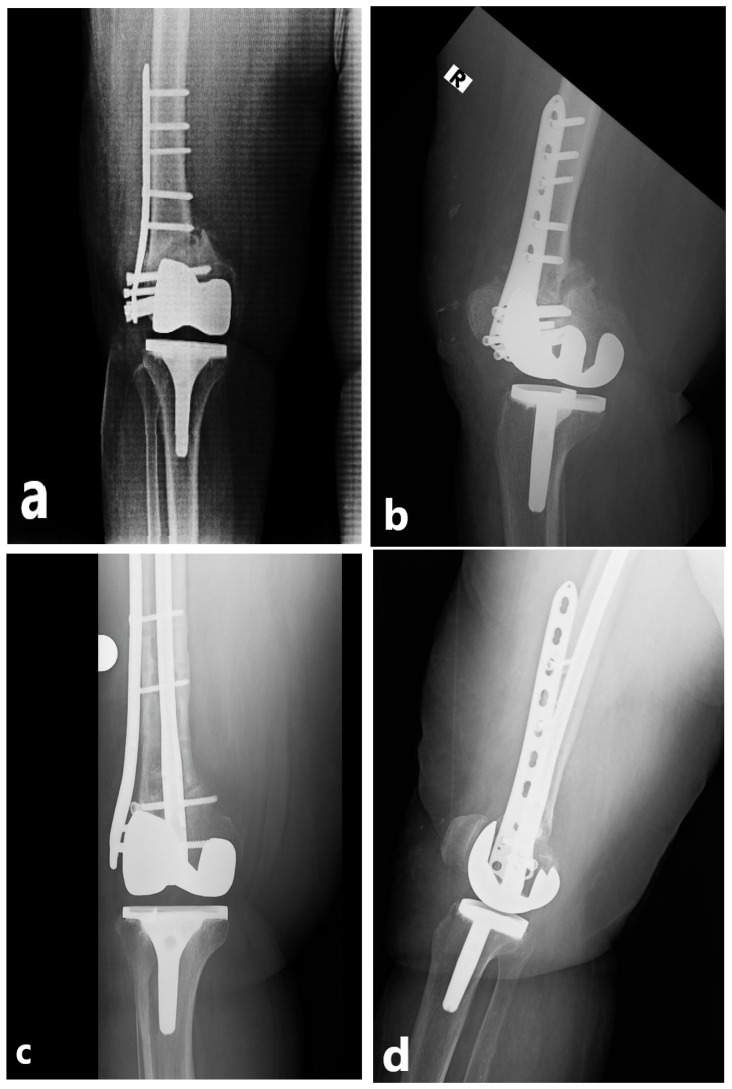
Radiographic images of a 72-year-old female. (**a**,**b**) Anteroposterior and lateral views demonstrating implant failure after the initial fixation of a periprosthetic distal femur fracture around a total knee arthroplasty. (**c**,**d**) Anteroposterior and lateral radiographs at postoperative 3 months following revision with a nail–plate combination. The radiographic marker “R” indicates the right extremity.

**Figure 3:**
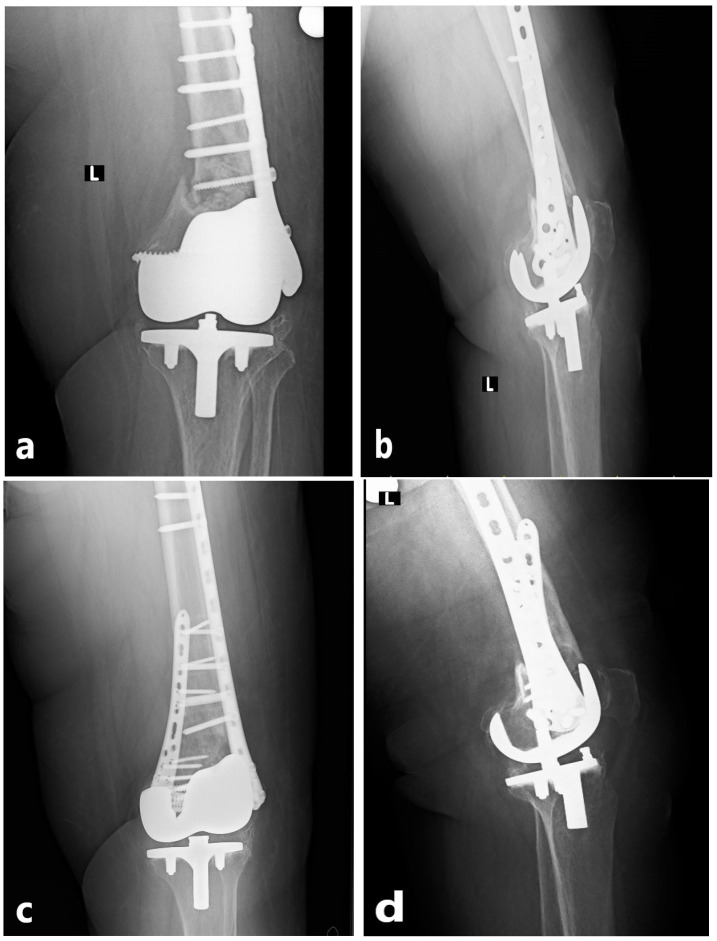
Radiographic images of a 76-year-old female patient. (**a**,**b**) Anteroposterior and lateral views demonstrating implant failure after the initial fixation of a periprosthetic distal femur fracture. (**c**,**d**) Anteroposterior and lateral radiographs at postoperative 3 months following revision with double-plate fixation. The radiographic marker “L” indicates the left extremity.

All procedures were performed by the same orthopedic trauma team, each surgeon having a minimum of 5 years of experience in periprosthetic fracture surgery, using standardized techniques. Patients were operated in the supine position under spinal or general anesthesia, with the knee slightly flexed and the foot free. Prophylactic antibiotics were administered preoperatively. During revision surgery, all failed implants from the initial fixation were removed, the fracture site was reassessed, and stability was restored with the chosen technique. All primary fixations consisted of anatomical distal femoral lateral locking plates, with supplemental cable fixation used in selected cases for additional metaphyseal or comminuted fragment support. During revision surgery, the previous lateral incision was routinely utilized. The decision regarding the surgical method was left to the operating surgeon. In the NPC group, a retrograde intramedullary femoral nail (ODI, Tampa, FL, USA) was combined with a distal femoral anatomic lateral locking plate (Zimed^®^, Gaziantep, Turkey). A small medial parapatellar incision was made to access the intercondylar notch, the canal was prepared with a guidewire, and an appropriately sized nail was inserted. Stabilization of the distal fragment was achieved with multiple interlocking screws, followed by placement of the lateral locking plate through a separate incision and fixation with screws aligned with the nail. This technique aimed to achieve both intramedullary load sharing and cortical support (Figure 2). In the NPC group, the length of the lateral plate was selected to allow adequate overlap with the intramedullary nail and to achieve stable fixation of the proximal fragment, typically ensuring fixation with at least three bicortical screws proximal to the fracture site. In the DP group, an anatomical distal femoral lateral locking plate (Zimed^®^, Gaziantep, Turkey) was applied through a lateral incision, while either an anatomical distal femoral locking plate or a standard 3.5 mm locking plate (Zimed^®^, Gaziantep, Turkey) was used on the medial side depending on fracture morphology and bone quality, thereby providing stable dual-column fixation (Figure 3). Bone grafting was not used in any patient. Anatomical reduction was confirmed fluoroscopically, hemostasis was achieved, a hemovac drain was placed, and wounds were closed in layers. Postoperatively, all patients received prophylactic antibiotics within the first 24 h and low-molecular-weight heparin for venous thromboembolism prophylaxis. Hemovac drains were typically removed within 24–48 h. Passive knee motion was initiated early to prevent stiffness and muscle atrophy. Mobilization was individualized according to fixation stability. Patients with stable fixation were allowed partial weight-bearing with crutches during the first postoperative week, while those with osteoporotic or unstable fractures followed a gradual loading protocol, typically achieving full weight-bearing by 6–8 weeks. Postoperative rehabilitation and weight-bearing protocols were standardized across both groups and individualized according to fixation stability, independent of the fixation technique used. Routine follow-up visits were scheduled at 2 weeks, 6 weeks, 3 months, 6 months, and 12 months, with annual follow-up thereafter. Each visit included clinical examination, radiographic assessment, and screening for complications.

### 2.3. Assessment Parameters

Demographic parameters included age, sex, body mass index (BMI), American Society of Anesthesiologists (ASA) classification, Charlson Comorbidity Index (CCI), smoking status, T-score by dual-energy X-ray absorptiometry (DEXA), presence of osteoporosis, and Arbeitsgemeinschaft für Osteosynthesefragen/Orthopaedic Trauma Association (AO/OTA) classification [10,11,12]. The CCI was calculated based on comorbidities recorded in patient files, following the original definition, which assigns scores from 1 to 6 to 19 chronic conditions, with the total score reflecting comorbidity burden [10]. Osteoporosis was defined as a T-score ≤ −2.5 or current use of anti-osteoporotic therapy, according to World Health Organization criteria [11]. AO/OTA fracture classification was independently assessed by two experienced orthopedic surgeons; disagreements were resolved by consensus. Perioperative parameters included operative time, intraoperative blood loss, fluoroscopy time, hospital stay, and transfusion requirement. Operative time was measured from skin incision to final closure. Blood loss was calculated by subtracting irrigation volume from suction canister output and by weighing surgical sponges [13]. Fluoroscopy time was defined as total radiation time in minutes. Hospital stay was calculated as the number of days from surgery to discharge. Transfusion was indicated for hemoglobin < 8 g/dL or symptomatic anemia, and all transfusions were recorded [14]. The type of implant used in the primary fixation was also retrieved from patient records and included in the analysis. Radiographic evaluation was performed using standardized anteroposterior and lateral femoral radiographs. Union was defined as the presence of tricortical bridging callus across the fracture site [15]. Union time was measured from the date of surgery to the date of radiographic union. Clinical evaluation included functional outcomes measured by the Knee Society Score (KSS), Western Ontario and McMaster Universities Osteoarthritis Index (WOMAC), and the SF-36 health survey (physical and mental component scores) [16]. All clinical outcome measures were assessed at the final follow-up visit. Complications recorded were infection, thromboembolism, neurovascular injury, implant failure, nonunion, malalignment, need for reoperation, and one-year mortality. Nonunion was defined as absence of radiographic progression or implant failure at 6 months. Malalignment was defined as >5° varus/valgus in the coronal plane or >10° flexion/extension in the sagittal plane [17]. Clinical and radiological assessments were independently performed by two orthopedic surgeons not involved in patient treatment and blinded to group allocation, thereby minimizing observer bias and enhancing reliability.

### 2.4. Statistical Analysis

All statistical analyses were performed using SPSS Statistics, Version 27.0 (IBM Corp., Armonk, NY, USA). Normality of data distribution was assessed with the Kolmogorov–Smirnov test. Continuous variables with normal distribution were compared using the independent-samples *t*-test, while those not normally distributed were analyzed with the Mann–Whitney U test. Categorical variables were compared using Pearson’s chi-square, Fisher’s exact, or Monte Carlo simulation tests. Prior to the study, an a priori power analysis was conducted based on previously published data evaluating differences in union time between fixation techniques in complex distal femur fractures, assuming a medium-to-large effect size (Cohen’s d = 0.7), which indicated that a minimum sample size of 72 patients would provide 90% statistical power. Post hoc power analysis confirmed that a medium-to-large effect size was achieved for parameters found to be statistically significant. Continuous variables are presented as mean ± standard deviation, median, and interquartile range, while categorical variables are reported as number and percentage. A *p*-value < 0.05 was considered statistically significant.

## 3. Results

The demographic characteristics are presented in Table 1. Age, sex distribution, BMI, ASA classification, CCI, smoking status, DEXA-measured T-scores, presence of osteoporosis, and AO/OTA fracture classification were similar between the two groups (Table 1).

When surgical parameters were compared, the DP group had significantly longer operative time, greater intraoperative blood loss, longer fluoroscopy time, and longer hospital stay (all *p* < 0.001). There were no significant differences between groups in transfusion requirement, follow-up duration, time to implant failure, or type of implant used in the primary surgery (Table 2).

Radiological and clinical outcomes showed that union time was significantly shorter in the NPC group (15.4 ± 2.8 weeks vs. 18.8 ± 3.5 weeks, *p* < 0.001). Functional outcomes, including WOMAC, SF-36 physical and mental component scores, and KSS, did not differ significantly between groups (Table 3).

Complications and mortality are summarized in Table 4. Rates of infection, thromboembolism, neurovascular injury, intensive care admission, nonunion, malalignment, implant failure, revision surgery, and one-year mortality showed no statistically significant differences between groups. Although complication rates were numerically higher in the DP group, this did not reach statistical significance. Implant failure was the most common complication, observed in 3.7% of NPC cases and 13.3% of DP cases. One-year mortality was calculated as 7.4% in the NPC group and 11.1% in the DP group. Postoperative infections were managed with surgical debridement and targeted antibiotic therapy. Cases of nonunion and implant failure were treated with revision fixation using either NPC or DP techniques, depending on fracture characteristics and bone quality.

## 4. Discussion

This study compared the NPC and DP fixation techniques in revision surgery for periprosthetic distal femur fractures following fixation failure. The findings demonstrate that the NPC technique provides clear advantages in terms of operative time, intraoperative blood loss, and union time, whereas functional outcomes and complication rates were comparable between the two methods.

Although several fixation strategies have been described for primary periprosthetic distal femur fractures, data focusing specifically on revision surgery after fixation failure remain limited [18]. The DP technique has long been favored for unstable fractures and those with metaphyseal–diaphyseal extension due to its high rigidity [4,19,20,21]. Ricci et al. reported that double plating provides early stability but is associated with longer operative time and greater blood loss [22]. Henderson et al. observed complication rates exceeding 20%, highlighting infection and implant failure as key concerns [23]. Stoffel et al. demonstrated in a biomechanical study that while double plating improves resistance to varus forces, its high rigidity may impair biological healing [24]. In the present series, operative time and blood loss were also higher in the DP group. The absence of significant differences in complication rates may indicate true clinical equivalence between the two fixation techniques. The relative homogeneity of the patient cohort and the fact that all procedures were performed by an experienced team within a single institution reduce potential bias and support the interpretation that the observed similarity in outcomes is related to the techniques themselves rather than to confounding factors. In addition, primary fixation failure was mainly mechanical in nature and included implant breakage, loss of fixation with secondary displacement, screw loosening or pull-out, and nonunion. In most cases, failure appeared to be multifactorial and developed progressively under physiological mechanical loading rather than as a result of an acute traumatic event.

In the present study, double-plate fixation was more frequently used in AO/OTA 33-C3 fractures compared with the nail–plate combination. Given that 33-C3 fractures represent the most complex and highly comminuted distal femoral patterns, this distribution likely reflects surgeon preference in demanding fracture configurations. In such cases, double plating is commonly perceived to provide superior control of fragment reduction and increased construct rigidity, particularly in the presence of severe metaphyseal comminution and segmental instability. This preferential selection represents real-world surgical decision-making rather than a predefined allocation strategy. Although this imbalance may introduce a degree of selection bias, it also indicates that double plating was more often reserved for the most challenging fracture patterns. Importantly, despite the higher fracture complexity in the DP group, overall complication rates and functional outcomes remained comparable between techniques in the present cohort.

The NPC technique has attracted increasing attention over the past decade, with several studies demonstrating both biomechanical and clinical advantages [1,8,25,26]. Hussain et al. showed in cadaveric models that NPC provides superior torsional and axial stability in osteoporotic bone [27]. Clinical series by Fulkerson et al. and Ehlinger et al. reported shorter operative time, reduced blood loss, and complication rates comparable to DP fixation [28,29]. The present results are consistent with these reports, as the NPC group showed a shorter mean union time by approximately three weeks. This finding may be explained by the physiologic load transfer achieved through intramedullary load sharing combined with lateral plate support.

The similarity in functional outcomes is in line with previous reports but requires careful interpretation. Periprosthetic distal femur fractures typically occur in elderly, osteoporotic patients with substantial comorbidity burden, in whom recovery of function is primarily determined by age and systemic conditions rather than fixation technique [29,30]. Therefore, perioperative and radiological differences between techniques may not directly translate into long-term functional scores. In the present study, WOMAC, KSS, and SF-36 scores were comparable, suggesting that patient-related factors exert a stronger influence on functional recovery than surgical method. Nevertheless, the shorter operative time, lower blood loss, and faster union associated with NPC represent meaningful perioperative advantages that may indirectly reduce postoperative complications and mortality, especially in frail, high-risk patients [6,26].

Comprehensive data on revision surgery after failed fixation of periprosthetic distal femur fractures remain scarce [18]. This study contributes to this limited body of evidence by demonstrating that NPC reduces surgical burden, shortens union time, and provides reliable outcomes in revision settings. Although DP fixation offers mechanical rigidity, previous reports have linked it to longer surgery, increased blood loss, and higher risk of soft-tissue complications [5,19]. In contrast, NPC provides a less invasive approach without extensive dissection, which may be particularly advantageous in elderly or medically high-risk patients. By reinforcing the biomechanical rationale with clinical data, these findings help inform surgical decision-making.

Several limitations should be acknowledged. First, the retrospective design carries inherent risks of selection bias and incomplete records. The groups were not fully balanced in terms of AO/OTA fracture patterns, and the absence of randomization together with surgeon-dependent implant selection introduces methodological heterogeneity. The mean follow-up of 23 months provides midterm results but does not address long-term implant survival, risk of re-revision, or conversion to arthroplasty. The limited sample size also reduces statistical power for rare events such as complications. The lack of Patient-Reported Outcome Measures (PROMs) restricts assessment from the patient perspective. Finally, cost-effectiveness and biomechanical parameters were not evaluated, limiting broader interpretation in terms of health economics and experimental validation. For these reasons, larger prospective multicenter studies are needed to confirm our findings.

## 5. Conclusions

This study demonstrates that the NPC provides distinct perioperative advantages over the DP technique in the revision surgery of periprosthetic distal femur fractures. The NPC group was associated with shorter operative time, reduced intraoperative blood loss, and faster radiological union, while functional outcomes and complication rates remained comparable between the two methods. These findings suggest that the NPC may represent a safer and more practical alternative.

## Figures and Tables

**Table 1:** Demographic characteristics of patients.

Variable	NPC Group (*n* = 27)	DP Group (*n* = 45)	*p*-Value
Age (years)	71.3 ± 5.6	72.2 ± 5.4	0.498
Sex (*n*)			
Female	18	33	0.547
Male	9	12	
BMI (kg/m^2^)	26.2 ± 3.5	27.5 ± 3.7	0.142
ASA score (*n*)			
II	8	13	0.963
III	12	19	
IV	7	13	
Charlson Comorbidity Index	4.7 ± 1.5	4.6 ± 1.5	0.721
Smoking (*n*)	3	11	0.166
T-score	−2.71 ± 0.40	−2.71 ± 0.39	0.939
AO/OTA classification (*n*)			
33A3	16	27	0.209
33C1	5	3	
33C2	4	5	
33C3	2	10	
Osteoporosis (*n*)	18	32	0.793

BMI: Body Mass Index, ASA: American Society of Anesthesiologists, AO/OTA: Arbeitsgemeinschaft für Osteosynthesefragen/Orthopaedic Trauma Association.

**Table 2:** Surgical parameters.

Variable	NPC Group (*n* = 27)	DP Group (*n* = 45)	*p*-Value
Surgery duration (min)	107.2 ± 14.2	133.9 ± 18.0	<0.001
Incision length (cm)	9.8 ± 1.9	13.9 ± 2.0	<0.001
Blood loss (mL)	411.9 ± 90.2	633.9 ± 151.2	<0.001
Fluoroscopy time (min)	2.9 ± 1.0	3.9 ± 1.4	0.001
Hospital stay (days)	6.9 ± 1.5	10.2 ± 3.3	<0.001
Follow-up time (months)	22.9 ± 5.6	23.6 ± 5.4	0.605
Time until implant failure (months)	10.3 ± 3.2	10.1 ± 3.6	0.861
Transfusion, (*n*)	10	14	0.606
Previous implant (*n*)			
Plate/cable	5	16	0.18
Locking plate	22	29	

**Table 3:** Clinical and radiological outcomes.

Variable	NPC Group (*n* = 27)	DP Group (*n* = 45)	*p*-Value
Union time (weeks)	15.4 ± 2.8	18.8 ± 3.5	<0.001
KSS	79.6 ± 7.5	76.5 ± 8.1	0.118
WOMAC	28.4 ± 9.2	33.6 ± 12.3	0.062
SF-36 Physical	65.4 ± 10.9	61.0 ± 11	0.106
SF-36 Mental	65.9 ± 11.5	61.4 ± 13.8	0.173

KSS: Knee Society Score, WOMAC: Western Ontario and McMaster Universities Osteoarthritis Index, SF-36: Short Form 36.

**Table 4:** Complications and mortality.

Variable	NPC Group (*n* = 27)	DP Group (*n* = 45)	*p*-Value
Infection	1	5	0.400
Thromboembolism	1	2	0.879
Neurovascular damage	0	2	0.525
Intensive Care Unit admission	1	4	0.402
Nonunion	2	6	0.701
Malalignment	2	6	0.701
Implant failure	1	6	0.244
Revision surgery	1	8	0.140
1-year mortality	4	6	0.861

## Data Availability

The original contributions presented in the study are included in the article, further inquiries can be directed to the corresponding authors.
